# Social Determinants of Health Documentation in Structured and Unstructured Clinical Data of Patients With Diabetes: Comparative Analysis

**DOI:** 10.2196/46159

**Published:** 2023-08-22

**Authors:** Shivani Mehta, Courtney R Lyles, Anna D Rubinsky, Kathryn E Kemper, Judith Auerbach, Urmimala Sarkar, Laura Gottlieb, William Brown III

**Affiliations:** 1 Department of Epidemiology and Biostatistics, University of California San Francisco, San Francisco, CA, United States; 2 Center for Vulnerable Populations, University of California San Francisco, San Francisco, CA, United States; 3 Department of Medicine, University of California San Francisco, San Francisco, CA, United States; 4 Bakar Computational Health Science Institute, University of California San Francisco, San Francisco, CA, United States; 5 Academic Research Services, Information Technology, University of California San Francisco, San Francisco, CA, United States; 6 Prevention Science, Department of Medicine, University of California San Francisco, San Francisco, CA, United States; 7 Department of Family and Community Medicine, University of California San Francisco, San Francisco, CA, United States; 8 Center for Digital Health Innovation, University of California San Francisco, San Francisco, CA, United States; 9 Center for AIDS Prevention Studies, Division of Prevention Science, University of California San Francisco, San Francisco, CA, United States

**Keywords:** natural language processing, diabetes mellitus, medical informatics applications, social determinants of health, NLP, machine learning, diabetes, diabetic, EHR, electronic health record, search engine, free text, unstructured data, text string

## Abstract

**Background:**

Electronic health records (EHRs) have yet to fully capture social determinants of health (SDOH) due to challenges such as nonexistent or inconsistent data capture tools across clinics, lack of time, and the burden of extra steps for the clinician. However, patient clinical notes (unstructured data) may be a better source of patient-related SDOH information.

**Objective:**

It is unclear how accurately EHR data reflect patients’ lived experience of SDOH. The manual process of retrieving SDOH information from clinical notes is time-consuming and not feasible. We leveraged two high-throughput tools to identify SDOH mappings to structured and unstructured patient data: PatientExploreR and Electronic Medical Record Search Engine (EMERSE).

**Methods:**

We included adult patients (≥18 years of age) receiving primary care for their diabetes at the University of California, San Francisco (UCSF), from January 1, 2018, to December 31, 2019. We used expert raters to develop a corpus using SDOH in the compendium as a knowledge base as targets for the natural language processing (NLP) text string mapping to find string stems, roots, and syntactic similarities in the clinical notes of patients with diabetes. We applied advanced built-in EMERSE NLP query parsers implemented with JavaCC.

**Results:**

We included 4283 adult patients receiving primary care for diabetes at UCSF. Our study revealed that SDOH may be more significant in the lives of patients with diabetes than is evident from structured data recorded on EHRs. With the application of EMERSE NLP rules, we uncovered additional information from patient clinical notes on problems related to social connectionsisolation, employment, financial insecurity, housing insecurity, food insecurity, education, and stress.

**Conclusions:**

We discovered more patient information related to SDOH in unstructured data than in structured data. The application of this technique and further investment in similar user-friendly tools and infrastructure to extract SDOH information from unstructured data may help to identify the range of social conditions that influence patients’ disease experiences and inform clinical decision-making.

## Introduction

There is growing recognition that addressing social determinants of health (SDOH)—the conditions in which people are born, grow, work, live, and age—in patient care is necessary for achieving optimal and equitable diabetes outcomes [1,2]. Prior evidence has shown that SDOH, particularly related to low socioeconomic status, affect disparities in the health care experience of patients with diabetes [3-5]. It is therefore imperative to better understand and intervene on SDOH to prevent negative clinical outcomes for patients and other downstream diabetes health care burdens and disparities [6]. SDOH can impact health equity in both a positive and negative way, thus leading to a gradient of health outcomes [7]. In this study, we focused on SDOH as a social risk factor on health outcomes.

Electronic health records (EHRs) are now becoming a resource to understand patients’ SDOH context in ways that could inform clinical practice. However, it remains unclear how accurately EHR data reflect patients’ lived experience of SDOH. Historically, EHRs have yet to fully capture SDOH due to challenges such as nonexistent or inconsistent data capture tools across clinics, lack of time and training, the burden of extra steps for the clinician, and the need for manual input, which can be a slow process [8]. Although structured data fields in EHRs for screening SDOH using *International Classification of Diseases (ICD)* codes have become more widespread, these are often not used by clinicians [9]. A better source of SDOH data from the EHR may be unstructured clinical notes, which provide qualitative detail beyond what is captured in structured data fields.

SDOH embedded in clinical notes could be captured quickly using natural language processing (NLP), but a corpus is hard to generate, and data access can be challenging, often requiring advanced programming skills. Novel and innovative high-throughput tools that automate and streamline the process of extracting SDOH data from clinical notes would prove useful to researchers and clinicians without advanced programming skills. Additionally, creating a high-throughput method of identifying SDOH mappings to structured and unstructured patient data has the potential to reduce physician charting burden and improve SDOH data in the EHR.

In 2018, researchers from the University of California, San Francisco (UCSF) created the Compendium of Medical Terminology Codes for Social Risk Factors that maps SDOH to existing ICD codes [10] (referred to hereafter as SDOH ICD Compendium or Compendium). The Compendium contains codes related to 20 SDOH-related risk and resilience factors from four medical vocabularies (LOINC, SNOMED CT, *International Classification of Diseases, Tenth Revision, Clinical Modification [ICD-10-CM]*, and Current Procedural Terminology) [10]. The Compendium allows us to identify existing codes related to social risk factors and their ontology.

In this study, we additionally leveraged two high-throughput tools for identifying SDOH mappings to structured (ICD codes) and unstructured (clinical notes) patient data: PatientExploreR and Electronic Medical Record Search Engine (EMERSE) [11]. We used these existing tools to first identify a cohort of patients within the EHR and explore the structured SDOH ICD Compendium codes (within PatientExploreR) and then to explore textual/unstructured data within the notes of the same patient population using the EMERSE NLP platform (grounded in the terminology from the SDOH ICD Compendium). This allowed us to identify and compare SDOH documentation in both structured and unstructured EHR data in records from patients with diabetes. Our working hypothesis was that these tools would reveal greater information about SDOH among these patients—through the mining of unstructured data—than is captured solely by structured data.

## Methods

### Deidentified Clinical Data Warehouse and PatientExploreR

**Figure 1.**
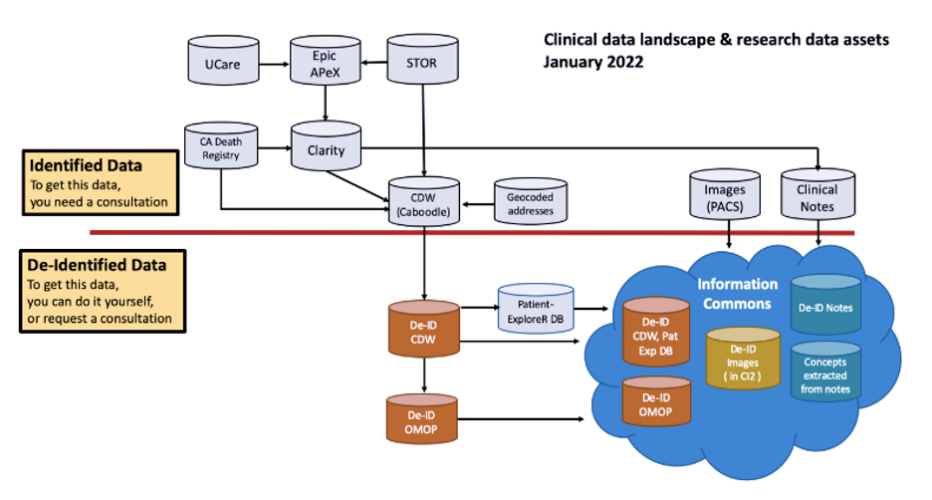
Clinical data landscape. CDW: Clinical Data Warehouse; DB: database; De-ID: deidentified; OMOP: Observational Medical Outcomes Partnership; PACS: picture archiving and communication system; Pat Exp: PatientExploreR.

UCSF EHR data were extracted using the SQL-based deidentified Clinical Data Warehouse (De-ID CDW) and PatientExploreR. The De-ID CDW is a deidentified database copy of high-value UCSF EHR data (Figure 1). De-ID CDW files are updated monthly, are not subject to Health Insurance Portability and Accountability Act (HIPAA) restrictions on research use, do not require institutional review board approval or an honest broker intermediary, and are available to the UCSF research community at no charge.

The De-ID CDW includes data from UCSF’s Epic-based EHR tool and historical EHR data prior to Epic adoption. The De-ID CDW includes the following data elements from the Epic EHR at UCSF Health: patient demographic and geographic information, allergies, billing, coverage, diagnoses, encounters, immunizations, lab, medication orders, procedure orders, providers, clinical notes, and vitals.

PatientExploreR is a user-friendly R Shiny application that enables rule-based mining of structured, clinical, patient-level interactive dynamic reports using Boolean operators and provides auto-generated visualization of clinical data. PatientExploreR’s data pipeline comes from the De-ID CDW, and exploration of the EHR data requires no advanced programming skills, as PatientExploreR data can be queried and extracted in a web-based format.

### Data Inclusion Criteria

For this study, we queried PatientExploreR to identify all adult patients (≥18 years of age) receiving primary care services for diabetes between January 1, 2018, to December 31, 2019. Primary care patients were defined as those who had completed two office visits with a primary care department on different dates of service and who had a documented encounter diagnosis of diabetes (type I or type II; *ICD-10-CM*: E10, E11) [12]. We excluded patients who were receiving only specialist care for diabetes as there may be systematic differences in patients receiving specialist care compared to primary care. Additionally, a specialist’s documentation related to SDOH may differ from that of primary care physicians and be less generalizable [13].

### EMERSE NLP Methods

**Figure 2.**
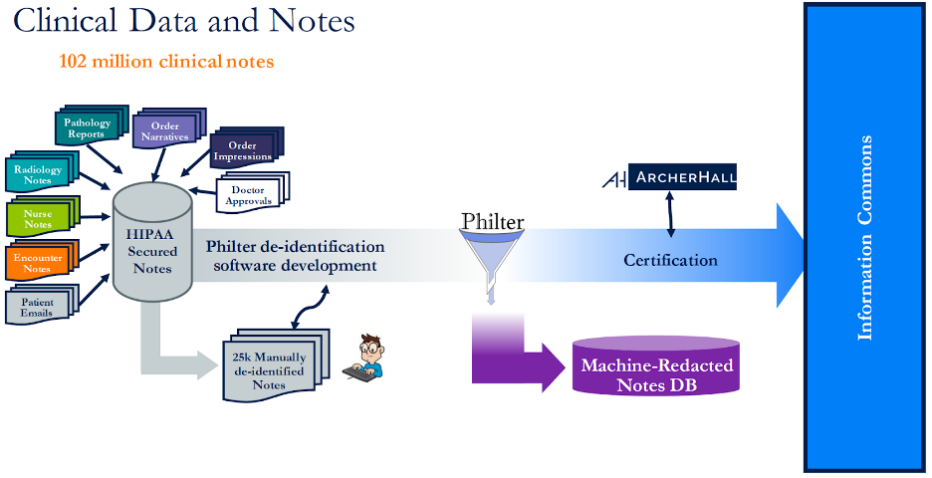
Process of Philter deidentification software for University of California, San Francisco, clinical notes. DB: database; HIPAA: Health Insurance Portability and Accountability Act.

**Figure 3.**
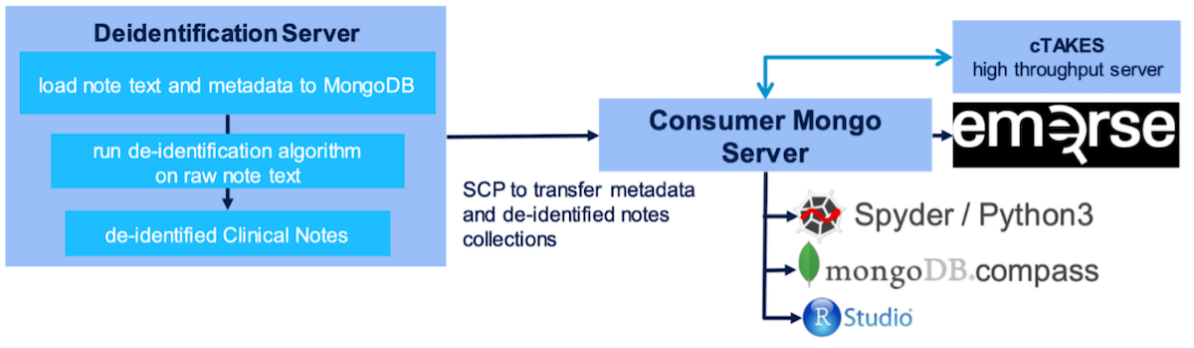
Schematic illustrating machine-redacted clinical notes data flow. SCP: secure copy protocol.

EMERSE clinical notes are deidentified through automated machine redaction using a protected health information filter [14] (Figure 2). A visualization of the machine-redacted clinical notes data flow is provided in Figure 3. We used EMERSE to extract clinical notes through a user-friendly interface [11]. We applied advanced built-in NLP query parsers implemented with JavaCC. The Lucene package enabled us to create our own rule-based approach queries through an application programming interface and provided parsing, tokenization features, and proximity searches [15]. We included clinical notes categorized as progress notes, telephone encounters, history and physical examinations, and assessment and plan notes.

### Text Corpus for NLP

**Figure 4.**
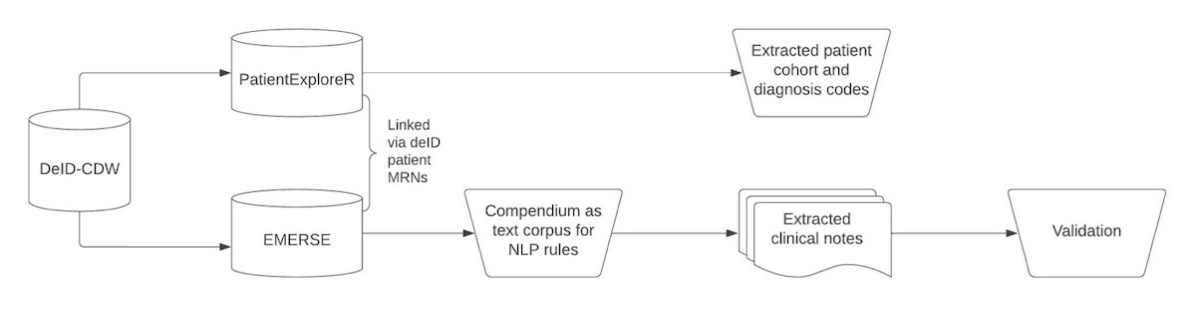
Study flow. CDW: Clinical Data Warehouse; DeID: deidentified; EMERSE: Electronic Medical Record Search Engine; MRN: medical record number; NLP: natural language processing.

For the same cohort of patients with diabetes identified within PatientExploreR, we then conducted a second exploration of SDOH documentation within the unstructured clinical notes (Figure 4). We linked the deidentified patient identifiers from PatientExploreR to the EMERSE platform, in which we were able to explore retracted clinical notes for the same patients during their primary care encounters within the same time period.

To compare the SDOH ICD Compendium codes from the structured EHR data to the unstructured clinical notes, we first transformed the Compendium into a set of textual search terms and concepts that would emerge within the free-text sections of the physician’s note. We used expert raters to develop a corpus using SDOH in the Compendium as a knowledge base as targets for the NLP text string mapping to find string stems, roots, and syntactic similarities in the clinical notes of patients with diabetes. To reduce false positives, we applied Boolean logic and proximity searches to create an exclusion term list within the NLP rule (Table 1). We determined a priori that the threshold to stop making changes to a rule was when it impacted less than 1% of the cohort.

**Table 1.** Natural language processing (NLP) rules.

SDOH^a^	NLP rule
Social connections/isolation	(“social isolation”~10th OR “socially isolated”~10th OR “feeling lonely”~10th OR “Loneliness” OR “isolation sad”~5) NOT (“no loneliness”~5 OR “not lonely”~5 OR “no social isolation” OR “denies loneliness”~4 OR “doesn't feel lonely” OR “isolation 14”~6 OR “can lead to loneliness and isolation” OR “isolation quarantine”~10th OR “isolation none”~5)
Employment	(unemploy* OR “job loss” OR “job fired”~10th OR “job worry”~10th OR “job ruined”~5 OR “job issues”~10th OR “problems at work” OR jobless* OR “does not get hired” OR “looking for work” OR “work fired”~10th OR “stressors work”~4 OR “out of work”)
Housing	((homeless* OR “housing instability” OR “unstable housing” OR evict* OR “shelter” OR “mold house”~10th OR “stressful home situation” OR “search for new place”) OR (“home safety” AND “home environment”)) NOT (“Homeless clients only” OR “volunteering homeless”~10th OR “would homeless”~5 OR “work homeless”~10th OR “homeless mother”~10th OR “homeless father”~10th OR “shelter in place” OR “shelter at home” OR “face tent” OR “oxygen tent”)
Food	(“food insecurity” OR “food insecure” OR “food pantry” OR “food stamp”) NOT (“FOOD INSECURITY: Negative” OR “Denies food insecurity”~10th OR “Food insecurity - worry” OR “Food secure” OR “No food insecurity” OR “Food insecure?” OR “No concerns raised re: food insecurity” OR “does not food pantry”~10)
Education	(Illitera* OR “lack of education” OR “poor education” OR “cannot read” OR “unable to read”) NOT (“label ripped”~3 OR “glucometer” OR “eyesight” OR “vision” OR “small print”)
Finance	(“Poverty” OR “low income” OR “no income” OR “financial difficulty” OR “financial difficulty” OR “financial difficulties” OR “financial issues” OR “financial burden” OR “financial assistance” OR “financial strain” OR “financial support” OR “financial need”) NOT (“not on file”, “if you qualify” OR “none” OR “doesn't qualify”~5 OR “resources”)
Stress	(“family stress”~5 OR "stressed” OR “stressful life”~5 OR “emotional stress” OR “headache stress”~5 OR “feels stressed”~5 OR “very stressed” OR “life stress”) NOT (score OR lab OR echo OR fracture OR myocardial OR perfusion OR exercise OR ecg OR test OR myocardial OR calculate OR ischemia OR ulcer OR induce OR “stressed importance” OR “stressed good”)

a SDOH: social determinants of health.

### Validation

Two independent reviewers (SM and JA) manually assessed the clinical notes for each SDOH domain to validate the classification performance of the NLP rule. One reviewer (SM) manually reviewed 100% of the clinical notes from each SDOH domain to validate the NLP rule’s classification performance. Reviewers tagged the clinical note as a true positive if the narrative had at least one mention of the social risk factor associated with the respective SDOH domain (Table 2).

**Table 2.** Examples of true-positive and false-positive terminologies.

SDOH^a^ domain	True-positive terminology	False-positive terminology
Social connections/ isolation	“Pt has difficulties with her mother and social isolation”	“She is deeply concerned about her son’s social isolation”
Employment	“Currently unemployed”	“Unemployed son”
Housing	“Pt with unstable housing situation, homeless”	“Volunteers at the animal shelter”
Food insecurity	“Diet remains problem with severe food insecurity”	“Food insecurity: none”
Education	“Never went to school and cannot read”	“Cannot read fine print as well, notes older glasses work better at near”
Finance	“Pt has low income subsidy, financial difficulties”	“Withdrawn cognition: poverty of thought”
Stress	“Feeling very stressed”	“Wife is stressed or complaining”

a SDOH: social determinants of health.

A second reviewer (JA) conducted a 2-step validation process. First, all the patients with clinical notes tagged “positive” for SDOH social risk factors by SM were reviewed by JA to ascertain the observed proportional agreement. Second, we randomly sampled 10% of clinical notes from each SDOH domain to ascertain the interrater agreement between SM and JA.

### Statistical Analysis

We use the Center for Medicaid Services *ICD-10* Z code groupings to calculate the prevalence of patients with SDOH documented within their structured data [16]. Our first goal was to understand SDOH documentation discrepancies between structured and unstructured clinical notes. Our second goal was to use a user-friendly informatics tool, EMERSE, to develop an NLP rule for each SDOH domain that was able to identify patients with clinical notes containing documentation of the SDOH domains. As part of the validation process, we calculated the proportion of observed agreement and Cohen kappa between the two independent reviewers for each SDOH domain (Multimedia Appendix 1).

### Ethical Considerations

The institutional review board at the University of California San Francisco approved this study (IRB number: 18-25696).

## Results

We identified 4283 adult (≥18 years of age) patients with 30,288 clinical notes receiving primary care for their diabetes (type I or type II; *ICD-10-CM*: E10, E11) at UCSF from January 1, 2018, to December 31, 2019. In the structured data, 16 (0.38%) patients had *ICD-10* Z60 codes for social connectionsisolation, 14 (0.33%) patients for stress, 4 (0.09%) patients for employment insecurity, 26 (0.61%) patients for housing insecurity, 39 (0.91%) patients for food insecurity, 4 (0.09%) patients for problems related to education, and 4 (0.09%) patients for financial insecurity (Table 3).

**Table 3.** Prevalence of patients with SDOH documentation in structured and unstructured data.

SDOH^a^ (*ICD-10^b^* code)	Patients in structured data (n=4283), n (%)	Patients in unstructured data (n=4283), n (%)
Social connections/isolation (60.2, 60.4, 60.8)	16 (0.38)	197 (4.60)
Employment (56.0, 56.1, 56.2, 56.89, 56.9)	4 (0.09)	197 (4.60)
Housing (59.0, 59.1, 59.8)	26 (0.61)	111 (2.59)
Food (59.4, 59.41)	39 (0.91)	102 (2.38)
Education (55.0, 55.1, 55.2, 55.3, 55.4, 55.8, 55.9)	4 (0.09)	35 (0.82)
Finance (59.5, 59.6, 59.7)	4 (0.09)	113 (2.64)
Stress (63.7, 63.79, 73.2, 73.3)	14 (0.33)	222 (5.18)

a SDOH: social determinants of health.

b *ICD-10: International Classification of Diseases, Tenth Revision*

### Social Connections/Isolation

Social connections/isolation (*ICD-10-CM* Z60) was defined as a lack of social connections or feelings of isolation or loneliness [10,16]. The NLP rule identified a total of 313 patients with documentation of social connections/isolation in their clinical notes. Of the 313 patients, 15 had a confirmed *ICD-10-CM* Z60 groupings diagnosis within their structured data, and 298 patients did not. A manual review of the clinical notes confirmed social connections/isolation problems for 197 (62.9%) of the 313 patients (Table 4).

**Table 4.** Manual review of clinical notes identified by the EMERSE NLP rules.

EMERSE^a^ NLP^b^ rule (+)	Manual review (+), n	Manual review (–), n	Total, n
Social connections/isolation	197	116	313
Employment	197	161	358
Housing	111	316	427
Food	102	55	157
Education	35	36	71
Finance	113	98	211
Stress	222	288	510

a EMERSE: Electronic Medical Record Search Engine.

b NLP: natural language processing.

### Employment Security

Employment insecurity (*ICD-10-CM* Z56) was defined as problems related to employment, unemployment, job loss, and work-related stressors [10,16]. The NLP rule identified a total of 358 patients with documentation of employment insecurity in their clinical notes. Of the 358 patients, 3 had a confirmed *ICD-10-CM* Z56 diagnosis and 355 patients did not. One patient did not have any clinical notes registered in the EMERSE system. Among 358 patients identified by the NLP rule, a manual review of the clinical notes confirmed problems related to employment for 197 (55%) patients (Table 4).

### Housing Security and Quality

This category included homelessness, problems with eviction, unsafe housing conditions (eg, mold), and unstable housing using the *ICD-10-CM* Z59 groupings [16]. The EMERSE NLP rule identified a total of 448 patients with documentation of housing insecurity/poor quality in their clinical notes. Of the 448 patients, 23 had confirmed Z59 diagnosis in their structured data and 425 patients did not. Among the 448 patients identified by the NLP rule, a manual review of the clinical notes confirmed problems with housing security and quality for 111 (24.8%) patients (Table 4).

### Food Security

Food insecurity (*ICD-10-CM* Z59.4) was defined as a lack of adequate food or intermittent access to food [10,16]. The NLP rule identified a total of 157 patients with documentation of food insecurity in their clinical notes. Of the 157 patients, 39 had a confirmed *ICD-10-CM* Z59.4 or *ICD-10-CM* Z59.41 diagnosis code in their structured data and 118 patients did not. Among 118 patients identified by the NLP rule, a manual review of the clinical notes confirmed food insecurity for 102 (65%) patients (Table 4).

### Education

The education category included patients with problems related to education, unable to read/write, or no formal education using the *ICD-10-CM* Z55 grouping [16]. The NLP rule identified a total of 71 patients with documentation of problems related to education in their clinical notes. Of the 71 patients, 4 had a confirmed *ICD-10* Z55 diagnosis code in their structured data and 67 did not. Among the 71 patients identified by the NLP rule, a manual review of the clinical notes confirmed problems related to education for 35 (49.3%) patients (Table 4).

### Finance

Financial insecurity (*ICD-10* Z59.5) was defined as patients reporting financial burdens, low income, poverty, or no income [10,16]. The NLP identified a total of 211 patients with documentation of financial insecurity in their clinical notes. Of the 211 patients, 4 had a confirmed *ICD-10* Z59.5, *ICD-10* Z59.6, or *ICD-10* Z59.7 diagnosis code in their structured data and 207 did not. Among the 211 patients identified by the NLP rule, a manual review of the clinical notes confirmed financial insecurity for 113 (53.6%) patients (Table 4).

### Stress

Stress was defined as the lack of relaxation and leisure, and difficulties with life management [10,16]. The NLP rule identified a total of 510 patients with documentation of stress in their clinical notes. Of the 510 patients, 11 had a confirmed *ICD-10* Z63.7, *ICD-10* Z63.79, *ICD-10* Z73.2, or *ICD-10* Z73.3 diagnosis code in their structured data and 499 did not. Among the 510 patients identified by the NLP rule, a manual review of the clinical notes confirmed stress for 222 (43.5%) patients (Table 4).

### Interrater Reliability

Observed proportional agreement between both reviewers ranged between 0.98 to 1 for the SDOH domains. The observed proportional agreement refers to the clinical notes in which both reviewers one and two have flagged as a positive for a social risk factor. Cohen kappa ranged from 0.21 to 1 (Multimedia Appendix 1). The validation process allowed us to understand the performance of the NLP rule’s classification. Overall, we discovered how much more the unstructured data yields about a patient’s SDOH in comparison to structured data.

## Discussion

### Findings

We included seven SDOH domains—social connections/isolation, problems related to employment, financial insecurity, housing insecurity, food insecurity, education, and stress—and conducted a manual review of clinical notes to validate the SDOH identification. Our study identified a greater proportion of individuals with diabetes who have an SDOH documented in their EHR when including clinical note data instead of structured data fields alone. In our sample, clinicians frequently captured information in their clinical notes about SDOH in the daily lives of their patients with diabetes, but they did not transfer it to the structured data field on the record, which is a core implementation consideration as the federal government and other agencies are looking to incentivize SDOH screening in the near future [17]. These documentation gaps may contribute to an underestimation of the overall impact of social (including material) and psychological factors on the health outcomes of people with diabetes that contribute to ongoing health disparities.

To the extent that information about SDOH is already being captured by clinicians in unstructured fields, informatics tools like NLP might be used to decrease new clinician structured field documentation burdens. The identification and classification of patients with SDOH using NLP methods is a complex process that involves the understanding of clinical note semantics, lexicon development, categorization, and manual validation.

There was a wide variation in the prevalence of SDOH elements in the unstructured data versus the structured data. The range of variation in the unstructured data depended on the SDOH domain—from 111 (24.8%) patients for housing insecurity compared to 197 (62.9%) patients for social connections/isolation. The findings highlight that future descriptive research should combine the usage of structured and unstructured data.

### Comparability

Our study findings are consistent with prior studies that found that EHR structured data underestimates SDOH. These studies found that less than 1% of cohorts had respective *ICD-10-CM* diagnosis codes for SDOH [18-21] documentation. Previous studies have shown that documentation about SDOH such as housing insecurity or lack of social connections or isolation, is 2-fold higher in unstructured data than in structured data [21-24]. However, none of these studies focused on patients with chronic health conditions like diabetes.

### Strengths and Limitations

To our knowledge, this is the first study to use PatientExploreR and EMERSE, two high-throughput tools, to identify SDOH mapping in structured and unstructured data for a population of patients with a specific chronic health condition. Neither tool requires users to have prior expertise in programming skills, which makes it accessible to a wider audience of clinicians and researchers. We used the compendium of medical terminology codes for social risk factors as a new data source to generate a corpus. The wider application of this adaptable technique may help to more robustly identify social factors that influence disease management and outcomes for a range of diseases and conditions and inform clinical decision-making.

There are several limitations of this study. This study was conducted using patient-level data from the UCSF medical system, which may limit external validity to the general population of patients with diabetes [25]. However, future work includes validating our NLP rules for a different patient population within the UCSF medical system. It is important to note that our inclusion criteria required patients to have a diagnosis code for diabetes, and this may have missed patients who had diabetes detected via medications or laboratory testing. Although we validated the NLP rule classification performance by manually reviewing the clinical notes that EMERSE deemed as containing SDOH documentation, we were unable to manually validate the clinical notes that our NLP rule did not pick up. This is a limitation as we were unable to calculate sensitivity, specificity, and common metrics to understand our NLP rule’s performance. Our NLP rules did not perform well for the following SDOH domains as we identified more false positives than true positives: housing security and quality, financial insecurity, and stress. This warrants further optimization to understand how these SDOHs are characterized within the clinical notes. Some SDOH domains may be more nuanced in terms of the language providers use to note them. This study discovered many false positives from the cases identified by the EMERSE NLP rule. High rates of false positives warrant further optimization of our NLP rule and understanding semantic differences of how SDOH are characterized within patient clinical notes. Future work will focus on enhancing the NLP rule and significant curation. Given the descriptive nature of this study, we did not assess the effects of the temporality of a patient’s SDOH, but we conducted a chart review to validate and assess the patient history of SDOH to the extent possible in unstructured notes.

Despite these limitations, this method has proven useful for clinicians and researchers interested in high-throughput ways to capture additional SDOH information related to patients to inform clinical decision-making. The ability to identify patients who are at risk via a streamlined high-throughput method can prevent downstream health burdens of social risk factors. Future work could focus on developing a rule-based machine learning algorithm to create and refine NLP rules associated with the other SDOH domains (eg, inadequate access to health care, incarceration, safety, and transportation barriers). Additionally, it is important for future work to understand the semantic variations that are used to characterize SDOH in clinical notes. Future research in this area to understand whether the performance of the NLP rule differed by certain patient characteristics (eg, age, race, and sex) would be valuable.

### Conclusion

Using unstructured data of patients with diabetes via EMERSE, we discovered more patient information related to a set of SDOH than we identified using structured data alone. Application of this technique, and future investments in similar user-friendly tools and infrastructure for capturing information from unstructured EHR data, may help to identify the range of social conditions that influence patients’ disease experience and inform clinical decision-making. If these data lead to improvements in clinical care and connections to social services, they are likely to result in improved patient health outcomes and, ideally, contribute to reducing health disparities.

## Supplementary material

10.2196/46159 Multimedia Appendix 1 Contingency table for interrater agreement per social determinant of health domain.

## Abbreviations

DeID-CDW: deidentified Clinical Data Warehouse
EHR: electronic health record
EMERSE: Electronic Medical Record Search Engine
HIPAA: Health Insurance Portability and Accountability Act
*ICD*: *International Classification of Diseases*
*ICD-10-CM*: *International Classification of Diseases, Tenth Revision, Clinical Modification*
NLP: natural language processing
SDOH: social determinants of health
UCSF: University of California, San Francisco
