# Supplementary material for: Social Determinants of Health Documentation in Structured and Unstructured Clinical Data of Patients With Diabetes: Comparative Analysis
Source: JMIR Med Inform. 2023 Aug 22;11:e46159. doi: 10.2196/46159 (PMC10466443; doi:10.2196/46159)
Supplement: Multimedia Appendix 1 — Contingency table for interrater agreement per social determinant of health domain. [file medinform-v11-e46159-s001.docx]

**Supplementary tables**

S1. Contingency table for inter-rater agreement per SDOH domain

| **SOCIAL CONNECTIONS/ISOLATION (0.58)** | | | |
| --- | --- | --- | --- |
|  | Rater 2 Yes | Rater 2 No | Total |
| Rater 1 Yes | 18 | 0 | 18 |
| Rater 1 No | 6 | 7 | 13 |
| Total | 24 | 7 | 31 |
| **EMPLOYMENT (Cohen’s k: 0.78)** | | | |
|  | Rater 2 Yes | Rater 2 No | Total |
| Rater 1 Yes | 14 | 0 | 14 |
| Rater 1 No | 4 | 18 | 22 |
| Total | 18 | 18 | 36 |
| **HOUSING (Cohen’s k: 0.62)** | | | |
|  | Rater 2 Yes | Rater 2 No | Total |
| Rater 1 Yes | 12 | 0 | 12 |
| Rater 1 No | 8 | 23 | 31 |
| Total | 20 | 23 | 43 |
| **FOOD (Cohen’s k: 1)** | | | |
|  | Rater 2 Yes | Rater 2 No | Total |
| Rater 1 Yes | 9 | 0 | 9 |
| Rater 1 No | 0 | 4 | 4 |
| Total | 9 | 4 | 13 |
| **EDUCATION (Cohen’s k: 1)** | | | |
|  | Rater 2 Yes | Rater 2 No | Total |
| Rater 1 Yes | 3 | 0 | 3 |
| Rater 1 No | 0 | 4 | 4 |
| Total | 3 | 4 | 7 |
| **FINANCE (Cohen’s k: 0.21)** | | | |
|  | Rater 2 Yes | Rater 2 No | Total |
| Rater 1 Yes | 12 | 0 | 12 |
| Rater 1 No | 8 | 2 | 10 |
| Total | 20 | 2 | 22 |
| **STRESS (Cohen’s k: 0.80)** | | | |
|  | Rater 2 Yes | Rater 2 No | Total |
| Rater 1 Yes | 23 | 0 | 23 |
| Rater 1 No | 5 | 22 | 27 |
| Total | 28 | 22 | 50 |
